# On Training Efficiency and Computational Costs of a Feed Forward Neural Network: A Review

**DOI:** 10.1155/2015/818243

**Published:** 2015-08-31

**Authors:** Antonino Laudani, Gabriele Maria Lozito, Francesco Riganti Fulginei, Alessandro Salvini

**Affiliations:** Department of Engineering, Roma Tre University, Via Vito Volterra 62, 00146 Rome, Italy

## Abstract

A comprehensive review on the problem of choosing a suitable activation function for the hidden layer of a feed forward neural network has been widely investigated. Since the nonlinear component of a neural network is the main contributor to the network mapping capabilities, the different choices that may lead to enhanced performances, in terms of training, generalization, or computational costs, are analyzed, both in general-purpose and in embedded computing environments. Finally, a strategy to convert a network configuration between different activation functions without altering the network mapping capabilities will be presented.

## 1. Introduction

Neural networks (NNs) are generally accepted in literature as a versatile and powerful tool for nonlinear mapping of a generic *n*-dimensional nonlinear function. The mapping capabilities of a NN are strictly related to the nonlinear component found in the activation function (AF) of the neurons. Indeed, without the presence of a nonlinear activation function, the NN would be a simple linear interpolator. The most generic representation of a NN is a group of elementary processing units (neurons) characterized by a weighted connection to *n* other input units. The processing of the unit consists of a linear part, where the inputs are linearly combined through the weights values, and a nonlinear part, where the weighted combination of the inputs is passed through an activation function, which is usually a threshold/squashing function. The nonlinear part of a NN is completely separated from the linear combination of the weighted inputs, thus opening a large number of possibilities for the choice of an activation function. Given the representation of the elementary unit, the inner architecture of the NN expresses the way those units are connected between themselves and to the inputs and outputs of the NN itself. Numerous authors studied the mapping capabilities of a NN, according to the inner architecture. In particular, it has been proved that the simple feed forward architecture with a single layer [1] and multiple layer [2–6] can be used as universal approximator given mild assumptions on hidden layer. A feed forward neural network (FFNN) is a NN where the inner architecture is organized in subsequent layers of neurons, and the connections are made according to the following rules: every neuron of a layer is connected to all (and only) the neurons of the subsequent layer. This topology rule excludes backward connections, found in many recurrent NNs [7–11], and layer-skipping, found in particular NN architectures like Fully Connected Cascade (FCC) [12]. Another focal point is the a-dynamicity of the architecture: in a FFNN no memory or delay is allowed, thus making the network useful only to represent static models. On this particular matter, several studies showed that, even by lacking dynamic capabilities, a FFNN can be used to represent both the function mapping and its derivatives [13]. Nevertheless, the choice of a suitable activation function for a FFNN, and in general, for a NN, is subject to different criterions. The most common considered criterions are training efficiency and computational cost. The former is especially important in the occurrence that a NN is trained in a general-purpose computing environment (e.g., using Matlab); the latter is critical in embedded systems (e.g., microcontrollers and FPGA (Field Programmable Gate Array)) where computational resources are inherently limited. The core of this work will be to give a comprehensive overview of the possibilities available in literature concerning the activation function for FFNN. Extending some of the concepts shown in this work could be possible to dynamic NNs as well; however training algorithms for dynamic NNs are completely different from the one used for FFNN, and thus the reader is advised to exert a critical analysis on the matter. The paper will be structured as follows. In the first part of this survey, different works focusing on the training efficiency of a FFNN with a specific AF will be presented. In particular, three subareas will be investigated: first, the analytic AFs, which enclose all the variants on the squashing functions proposed in the classic literature [14, 15]; then, the fuzzy AFs, which exploit complex membership functions to achieve faster convergence during the training procedure; last, the adaptive AFs, which focus on shaping the NN nonlinear response to mimic as much as possible the mapping function properties. In the second part of this survey, the topic of computational efficiency will be explored through different works, focusing on different order approximations found in literature. In the third and last part of this survey, a method to transform FFNN weights and biases to change the AF of the hidden layer without need to retrain the network is presented. Conclusions will follow in the fourth part. An appendix, containing the relevant figures of merit reported by the authors in their work, closes this paper.

## 2. Activation Functions for Easy Training

### 2.1. Analytic Activation Functions

The commonly used backpropagation algorithm for FFNN training suffers from slow learning speed. One of the reasons for this drawback lies in the rule for the computation of the FFNN's weights correction matrix, which is calculated using the derivative of the activation function for the FFNN's neurons. The universal approximation theorem [1] states that one of the conditions for the FFNN to be a universal approximator is for the activation function to be bounded. For these reasons, most of the activation functions show a high derivative near the origin and a progressive flattening moving towards infinity. This means that, for neurons having a sum of weighted inputs very large in magnitude, learning rate will be very slow. A detailed comparison between different simple activation functions based on exponentials and logarithms can be found in [16], where the authors investigate the learning rate and convergence speed on a character recognition problem and the classic XOR classification problem, proposing the use of the inverse tangent as a fast-learning activation function. The authors compare the training performance, in terms of Epochs required to learn the task, of the proposed inverse tangent function, against the classic sigmoid and hyperbolic tangent functions, and the novel logarithmic activation function found in [17], finding a considerable performance gain. In [18], the sigmoid activation function is modified by introducing the square of the argument, enhancing the mapping capabilities of the NN. In [19], two activation functions, one based on integration of the triangular function and one on the difference between two sigmoids (log-exponential), are proposed and compared through a barycentric plotting technique, which projects the mapping capabilities of the network in a hyper dimensional cube. The study has shown that log-exponential function has been slowly accelerated but it was effective in MLP network with backpropagation learning. In [20] a piecewise interpolation by means of cubic splines is used as an activation function, providing performances comparable to the sigmoid function with reduced computational costs. In [21], the proposed activation function is derived by Hermite orthonormal polynomials. The criterion is that every neuron in the hidden layer is characterized by a different AF, which is more complex for every neuron added. Through extensive simulations, the authors prove that such network shows great performance in comparison to analogous FFNN with identical sigmoid AFs. In [22], the authors propose a performance comparison between eight different AFs, including the stochastic AF and the novel “neural” activation function, obtained by the combination of a sinusoidal and sigmoid activation function. Two tests sets are used for comparison: breast cancer and thyroid diseases related data. The work shows that the sigmoid AFs yield, overall, the worst accuracy, and the hyperbolic tangent and the neural AF perform better on breast cancer dataset and thyroid disease dataset, respectively, pointing out the dataset dependence of the AF capabilities. The “neural” AF is investigated in [23] as well (in this work, it is referred to as “periodic”), where the NN is trained by the extended Kalman filter algorithm. The network is tested, against classic sigmoid and sinusoidal networks, in handwriting recognition, time series prediction, parity generation, and XOR mapping. The authors prove that the periodic function proposed outperforms both classic AFs in terms of training convergence. In [24], the authors suggest the combination of sigmoid and sinusoidal and Gaussian activation function, to exploit their independent space division properties. The authors compare the hybrid structure in a multifrequency signal classification problem, concluding that even if the combination of the three activation functions performs better than the sigmoid (in terms of convergence speed) and the Gaussian (in terms of noise rejection), the sinusoidal activation function by itself still achieves better results. Another work investigating an activation function based on sinusoidal modulation can be found in [25], where the authors propose a cosine modulated Gaussian function. The use of sinusoidal activation function is deeply investigated in [26], where the authors present a comprehensive comparison between eight different activation functions on eight different problems. Among other results, the Sinc activation function is proved as a valid alternative to the hyperbolic tangent, and the sinusoidal activation function has good training performance on small FFNNs. In Table 1, a summary of the different analytical AFs proposed in this paragraph is shown.

### 2.2. Fuzzy Logic

An approach often found in literature is to combine FFNN with fuzzy logic (FL) techniques to create fast converging FFNN. In [27], the authors define the hyperbolic tangent transfer using three different membership functions, defining in fact the classical activation function by means of the fuzzy logic methodology. The main advantage during the training phase is a low computational cost, achieved since weight updating is not always necessary. The performance validation is verified through a comparison between two identically initialized FFNNs, one with the hyperbolic tangent and one with the proposed activation function. The two FFNNs are tested on the problems of XOR gate, one-step-ahead prediction of chaotic time series, equalization of communication channels, and independent components analysis. The authors in [28] use a Type 2 Fuzzy Function, originally proposed in [29, 30], as the activation function for a FFNN, and compare the training performance on the classic XOR problem and induction motor speed prediction. An additional application of NN with fuzzy AFs can be found in [31], where the authors use the network for detection of epileptic seizure, processing and classifying EEG signals. In [32], the authors propose an analytic training method, noted as extreme machine learning (EML), for FFNNs with fuzzy AFs. The authors test the procedure on problems of medical diagnosis, image classification, and satellite image analysis.

### 2.3. Adaptive Strategies

Although the universal mapping capabilities of a FFNN have been proven, some assumptions on the activation function can be overlooked in favor of a more efficient training procedure. Indeed, it has been seen that a spectral similarity between the activation function and the desired mapping gives improved performance in terms of training [33]. The extreme version of this approach consists in having an activation function that is modified during the training procedure, creating in fact an ad hoc transfer function for neurons. Variations of this approach can be found for problems of biomedical signal processing [34], structural analysis [35], and data mining procedures [36]. The training algorithm for such networks requires taking into consideration the activation function adaptation, as well as the weights tuning. The authors in [37] propose a simple BP-like algorithm to train a NN with trainable AF and compare the training performance with a classic sigmoid activation function on both XOR problem and a nonlinear mapping.

## 3. Activation Functions for Fast Computation

The computational cost of a FFNN can be split into two main contributions. The first one is a linear cost, deriving from the operations needed to perform the sum of the weighted inputs of each neuron. The second one, nonlinear, is related to the computation of the activation function. In a computational environment, those operations are carried out considering a particular precision format for numbers. Considering the AF is usually limited in a small codomain, the use of integer arithmetic is unadvisable. Fixed-point and floating-point arithmetic are the most commonly used to compute the FFNN elementary operations. The linear part of the FFNN is straightforward: operations of products and sums are carried out by multipliers and adders (usually found in the floating-point unit (FPU) of an embedded device). The nonlinear part, featuring transcendental expressions, is carried out through complex arithmetic evaluations (IEEE 754 is the reference standard) that, in the end, still use elementary computational blocks like adders and multipliers. Addition and product with floating-point precision are complex and long operations, and the computational block that executes these operations often features pipeline architectures to speed up the arithmetic process. Although a careful optimization of the linear part is required to completely exploit pipeline capabilities [38], the ratio between the two costs shows, usually, that the linear quota of the operations is negligible when compared to the nonlinear part. In embedded environment, computational resources are scarce, in terms of both raw operations per second and available memory (or resources, for synthesizable digital circuits like FPGAs and ASICs). Since embedded applications usually require real-time interaction, the development of NN applications in embedded environments shows the largest contributions in terms of fast and light solutions for AFs computation. Three branches of approaches can be found in literature: PWL (piecewise linear) interpolation, LUT (Lookup-Table) interpolation, and higher order/hybrid techniques. The following part of this survey will present the different approaches found in literature, grouped under these three sets.

### 3.1. Lookup-Table Approximations

Approximation by LUT is the simplest approach that can be used to reduce the computational cost of a complex function. The idea is to store inside the memory (i.e., a table) samples from a subdomain of the function and access those instead of calculating the function. The table either can be preloaded in the embedded device (e.g., in the flash memory of a microcontroller) or could be calculated at run-time with values stored in the heap. Both alternatives are valid, and the choice is strictly application dependent. In the first case, the static approach occupies a memory section that is usually more available. In the second case, the LUT is saved in the RAM memory, which is generally smaller than the flash; however, in this case the LUT is adjustable in case a finer (or coarser) version is needed. A variation of the simple LUT is the RA-LUT (Range Addressable LUT), where each sample corresponds not only to a specific point in the domain, but to a neighborhood of the point. In [39] the authors propose a comparison between two FPGA architectures which uses floating-point accelerators based on RA-LUT to compute fast AFs. The first solution, refined from the one proposed in [40, 41], implements the NN on a soft processor and computes the AFs through a smartly spaced RA-LUT. The second solution is an arithmetic chain coordinated by a VHDL finite state machine. Both sigmoid and hyperbolic tangent RA-LUTs are investigated. In [42] an efficient approach for LUT address decoding is proposed for the hyperbolic tangent function. The decoder is based on logic expressions derived by mapping the LUT ranges into subdomains of the original function. The result obtained is LUT implementation that requires less hardware resources to be synthesized. A particular consideration was given to AFs derivatives in [43] where the authors studied the LUT and RA-LUT for on-chip learning applications. The authors conclude that the RA-LUT implementation yields on-chip training performances comparable with the Full-Precision Floating Point even introducing more than 10% precision degradation.

### 3.2. Piecewise Linear Approximations

Approximation through linear segments of a function can be easily carried out in embedded environment since, for every segment, the approximated value can be computed by one multiplication and one addition. In [44] the authors propose an implementation of a NN on two FPGA devices by Xilinx, the Virtex-5 and the Spartan 3. On both devices, the NN is implemented in VLSI language and as a high-level software running on microblaze soft processor. The authors conclude that although the implementation in VLSI is more complex, the parallel capabilities of the hardware neurons yield much higher performance overall. In [45] authors propose a low-error implementation of the sigmoid AF on a Virtex-4 FPGA device, with a comprehensive analysis on error minimization. To further push the precision versus speed tradeoff, in [46] the authors present a comparison between PWL and LUT techniques under both floating-point and fixed-point arithmetic, testing the approximation on a simple XOR problem. In [47] four different PWL techniques (three linear and one quadratic that will be discussed in the next section) are analyzed considering the hardware resources required for implementation, the errors caused by the approximation, the processing speed, and the power consumption. The techniques are all implemented using the System Generator, a Simulink/Matlab toolbox released by Xilinx. The first technique implemented by the authors is called A-Law approximation, which is based on a PWL approximation where every segment has a gradient expressed as a power of two, thus making it possible to replace multipliers with adders [48]. The second technique is the Alippi and Storti-Gajani approximation [49], based on a segmentation of the function in specific breakpoints where the value can be expressed as sum of power of two numbers. The third technique, called PLAN (Piecewise Linear Approximation of a Nonlinear Function), uses simple digital gate design to perform a direct transformation from input to output [50]. In this case, the shift/add operations, replacing the multiplications, were implemented with simple gate design that maps directly the input values to sigmoidal outputs. All the three techniques are compared together and against the classic LUT approach. The authors conclude that, overall, the best results are obtained through PLAN approximation. Approximation through PLAN method is used in [51] as well, where the authors propose a complete design in System Generator for on-chip learning through online backpropagation algorithm.

### 3.3. Hybrid and Higher Order Techniques

Hybrid techniques try to combine both LUT and PWL approximations to obtain a solution that yields a compromise between the accuracy of the PWL approximation and the speed of the LUT. Higher order techniques push the boundaries and try to represent the AF through higher order approximation (e.g., polynomial fitting). An intuitive approach is to use Taylor series expansion around origin and is used by [52, 53] at the 4th and 5th order, respectively. In [54, 55] the authors propose two approaches, one composed of a PWL approximation coupled with a RA-LUT and one composed of the PWL approximation with a combinatorial logic circuit. The authors compare the solutions with classic (Alippi, PLAN) PWL approximation focusing on resources required and accuracy degradation. Combination of LUT and PWL approximation is also used in [56, 57], where different authors investigate the approximation in fixed point for the synthesis of exponential and sigmoid AFs. In [39, 58] authors propose a piecewise II-degree polynomial approximation of the activation function for both the sigmoid and the hyperbolic tangent AFs. Performance, resources required, and precision degradation are compared to full-precision and RA-LUT solutions. In [59] authors propose a semiquadratic approximation of the AF (similar to the one proposed in [19]) and compare the embedded performances in terms of throughput and consumed area against simple PWL approximation. In [60] a simple 2nd-order AF, which features an origin transition similar to the hyperbolic tangent, is proposed. The digital complexity of this function is in the order of a binary product, since one of the two products required to obtain a 2nd-order function is performed by a binary shift. A similar 2nd-order approximation is proposed by Zhang et al. in [61] and is applied in [47] where the authors comprehensively compare it to 1st PWL techniques and LUT interpolations. In [62] the proposed approach is based on a two-piece linear interpolation of the activation function, later refined by correction factors stored in a LUT. This hybrid approach reduces the dynamic range of the data stored in the LUT, thus making it possible to save space on both the adder and the table itself.

### 3.4. Weight Transformation

Different papers shown in this survey pointed out advantages and drawbacks of using an AF instead of another one. Two very common AFs that are found in almost any comparison are the sigmoid activation function and the hyperbolic tangent activation function. Considering an embedded application, as the one suggested in [39], where the activation function is directly computed by a floating-point arithmetic chain of blocks, using a sigmoid AF instead of a hyperbolic tangent AF allows synthesizing the chain with less arithmetic units. However, as shown in several papers in Section 2, the low derivative of the sigmoid AF makes it a poor candidate for training purposes when compared to the hyperbolic tangent. In this final part of the survey, a set of transformation rules, for a single layer FFNN with arbitrary inputs and outputs, is proposed. The rules allow modifying weights and biases of a NN so that changing the hidden layer AF does not change the NN output:

(1) $$\begin{array}{c} B_{O}^{L}+\sum \dot{W_{O}^{L}}*\left(\frac{1}{1+e^{-\left\{B_{H}^{L}+\sum W_{H}^{L}*in\right\}}}\right)=B_{O}^{T}+\sum W_{O}^{T}*\left(\frac{2}{1+e^{-2\left\{B_{H}^{T}+\sum W_{H}^{T}*in\right\}}}-1\right), \end{array}$$

(2) $$\begin{array}{c} B_{O}^{L}+\sum \dot{W_{O}^{L}}*\left(\frac{1}{1+e^{-\left\{B_{H}^{L}+\sum W_{H}^{L}*in\right\}}}\right)=\left[B_{O}^{T}-\sum W_{O}^{T}\right]+\sum \left(2*W_{O}^{T}\right)*\left(\frac{1}{1+e^{-\left\{\left(2*B_{H}^{T}\right)+\sum \left({2*W}_{H}^{T}\right)*in\right\}}}\right), \end{array}$$

(3) $$\begin{array}{c} \left[B_{O}^{L}+\sum \frac{W_{O}^{L}}{2}\right]+\sum \left(\frac{\dot{W_{O}^{L}}}{2}\right)*\left(\frac{2}{1+e^{-\left\{\left(B_{H}^{L}/2\right)+\sum \left(W_{H}^{L}/2\right)*in\right\}}}\right)=B_{O}^{T}+\sum W_{O}^{T}*\left(\frac{2}{1+e^{-2\left\{B_{H}^{T}+\sum W_{H}^{T}*in\right\}}}-1\right). \end{array}$$

Right side of (1) shows the output for multiple inputs single output (MISO) FFNN with sigmoid activation function. Left side of (1) shows the output MISO FFNN with hyperbolic tangent activation function. In (2) and (3), right hand side and left hand side have been, respectively, rearranged to highlight the matrix relations that allow AF transformations. Indeed (1), (2), and (3) express the relations for a MISO FFNN, but it is straightforward to generalize a translation procedure valid for a MIMO network. Tables 2 and 3 allow an easy translation of weights and biases to switch between sigmoid and hyperbolic function AFs ([1] denotes a 1-by-*N* vector, where *N* is the number of output neurons). A possible strategy to exploit these relations would be to create a tanh based FFNN in a general-purpose environment, like Matlab. Then, after the FFNN has been trained, translate the weights in sigmoid form to obtain a FFNN that features a simpler AF.

## 4. Conclusions

The topic of the choice of a suitable activation function in a feed forward neural network was analyzed in this paper. Two aspects of the topic were investigated: the choice of an AF with fast training convergence and the choice of an AF with good computational performance.

The first aspect involved several works proposing alternative AFs, analytical or not, that featured quick and accurate convergence during the training process, while retaining generalization capabilities. Instead of finding a particular solution that performs better than the others, the aspect that was highlighted by several works is that the simple sigmoid functions that were introduced with the first FFNN, although granting the universal interpolation capabilities, are far from being the most efficient choice for a neural problem. Apart from this negative consideration, no deal-breaker conclusion can be drawn from the comparative analysis found in literature. Indeed, it has been shown that some preliminary analysis on the mapping function allows the choice of a “similar” AF that could perform better on a specific problem. However, this conclusion is a methodological consideration, rather than a position on a particular advantageous AF choice. On the other hand, the adaptive strategies proposed, which feature tunable AFs that can mimic some of the mapping function characteristics, suffer from a more complex and error prone training algorithm.

The second aspect involved both approximations of the classic AFs found in literature and brand new AFs specifically proposed for embedded solutions. The goal pursued by the works summarized in this section was to find a good deal between accuracy, computational costs, and memory footprint, in a limited resources environment like an embedded system. It is obvious that the direct use of a FFNN in embedded environment, without the aid of some kind of numerical approximation, is unfeasible for any real-time application. Even using a powerful computation system, featuring floating-point arithmetic units, the full-precision IEEE 745 compliant AF computation is a plain waste of resources. This is especially true for many embedded applications where the FFNN is used as a controller [63] that process data obtained from low precision sources [64] (e.g., a 10-Bit Analog-to-Digital Converter, which is found on numerous commercial microcontroller units). The point of the analysis was to show the wide range of possibilities that are available for the implementation, since, in this case, the particular choice of AFs is strictly application dependent. The optimum trade point between accuracy, throughput, and memory footprint cannot be generalized.

Unfortunately, the outcome of a successful NN solution of a problem is influenced by several aspects that cannot be separated from the AF choice. The choice of the training algorithm, for example, can go from simple backpropagation-like algorithms to heuristics assisted training, with all the possible choices in between. The suitable sizing of the hidden layer and the training/validation sets [65], the a priori simplification of the problem dimensionality [66], and use of pruning techniques like OBD (Optimal Brain Damage) [67] and OBS (Optimal Brain Surgeon) [68] to remove unimportant weights from a network all influence the outcome as well. And even if a standard procedure could be thought and proposed, there is no universally recognized benchmark that could be used to test and compare different solutions: as it can be seen from the works proposed, NN approach is tested on real world problems. This is not due to the lack of recognized benchmarks in literature [69] but rather due to the attitude of authors proposing a particular NN solution (whether it is a novel AF, an architecture, or a training algorithm), to test it only for a specific application. This hinders the transfer potential of the solution, enclosing it in the specific application it was thought for.

## Figures and Tables

**Table 1:** Analytic AFs.

Ref.	Name	Expression	Notes
[14, 15]	Step-like	$f\left(x\right)=\left\{\begin{array}{cc} a, & x\geq 0\\ b, & x<0 \end{array}\right.$	
	Linear	*f*(*x*) = *αx*	
	Saturated linear	$f\left(x\right)=\left\{\begin{array}{cc} 0, & x\leq 0\\ \alpha x, & 0<x<x_{m}\\ 1, & x\geq x_{m} \end{array}\right.$	Where to ensure continuity *x*_*m*_ is *α* ^−1^
	Sigmoid	$f\left(x\right)=\frac{1}{1+e^{-x}}$	
	Hyp. tangent	$f\left(x\right)=\frac{2}{1+e^{-2x}}-1$	

[16]	Arctangent	*f*(*x*) = tan^−1^⁡(*x*)	

[18]	Quadratic sigmoid function	$f\left(x\right)=\frac{1}{1+e^{\left(x^{2}-\theta \right)}}$	

[19, 22]	Logarithmic-Exponential	$f\left(x\right)=1-\frac{1}{2b}\ln\left[\frac{1+e^{a-cx+b}}{1+e^{a-cx-b}}\right]$	*a* is responsible for centering the function, *b* distances the sigmoid, and *c* is a coefficient that controls the slope

[19]	Triangular approximation	$f\left(x\right)=\left\{\begin{array}{cc} \mathrm{sign}\left(x\right)\times \left[0.5\times m_{1}\times {\left|x\right|}^{2}+c_{1}\times \left|x\right|+d_{1}\right], & 0\leq \left|x\right|\leq a\\ \mathrm{sign}\left(x\right)\times \left[0.5\times m_{2}\times {\left|x\right|}^{2}+c_{2}\times \left|x\right|+d_{2}\right], & a\leq \left|x\right|\leq b\\ \mathrm{sign}\left(x\right), & \text{otherwise} \end{array}\right.$	Where *m*_1_, *m*_2_, *c*_1_, *c*_2_, *a*, and *b* are −0.54324, −0.16957, 1, 0.42654, 1.52, and 2.57, respectively. *d*_1_ and *d*_2_ are 0.016 and 0.4519, respectively

[21]	Hermite polyn.	*f*(*x*) = *h*_*n*_(*x*) = *α*_*n*_ *H*_*n*_(*x*)*ϕ*(*x*), where $H_{n}\left(x\right)={\left(-1\right)}^{n}e^{x^{2}}\frac{d^{n}}{dx^{n}}\left(e^{-x^{2}}\right)$, *n* > 0, with *H*_0_ = 1 *α*_*n*_ = (*n*!)^−1/2^ *π* ^1/4^2^−(*n*−1)/2^ $\phi \left(x\right)=\frac{1}{\sqrt{2\pi }}e^{-x^{2}/2}$	

[22]	Gaussian	*f*(*x*) = *e* ^−(*x* − *c*)^2^/2*σ*^2^^	

[26]	PolyExp	*f*(*x*) = (*ax* ^2^ + *bx* + *c*) × *e* ^−*γx*^2^^	Where *a*, *b*, and *c* are tunable constants

[24, 26]	Wave	*f*(*x*) = (1 − *x* ^2^)*e* ^−*γx*^2^^	

[22, 23]	Neural	$f\left(x\right)=\frac{1}{1+e^{-S_{p}\sin\left(r_{p}\times x\right)}}$	Where *S*_*P*_ controls the slope while *r*_*P*_ governs the frequency of the resultant function

[23]	Sinusoidal	*f*(*x*) = 0.5(sin⁡(*r*_*s*_ × *x*) + 1)	Where *r*_*S*_ is a parameter related to the frequency of the sine function

[25]	CosGauss	*f*(*x*) = *e* ^−β*x*^2^^cos⁡(*γx*)	The parameter *β* in the function scales the gradient of the Gaussian function; meanwhile *γ* controls the frequency of repetition of the “hills”

[26]	Sinc	$f\left(x\right)=\left\{\begin{array}{cc} \frac{\sin\left(\pi x\right)}{x}, & x\neq 0\\ 1, & x=0 \end{array}\right.$	

[26]	SinCos	*f*(*x*) = *a*sin⁡(*p* × *x*) + *b*cos⁡(*q* × *x*)	Where *p* and *q* are frequencies and *a* and *b* are proportional parameters

**Table 2:** From sigmoid to tanh.

Hidden bias	[*B*_*H*_^*L*^] = 2 × [*B*_*H*_^*T*^]

Hidden weights	[*W*_*H*_^*L*^] = 2 × [*W*_*H*_^*T*^]

Output bias	[*B*_*O*_^*L*^] = [*B*_*O*_^*T*^] − [*W*_*O*_^*T*^][1]

Output weights	[*W*_*O*_^*L*^] = 2 × [*W*_*O*_^*T*^]

**Table 3:** From tanh to sigmoid.

Hidden bias	$\left[B_{H}^{T}\right]=\frac{1}{2}\times \left[B_{H}^{L}\right]$

Hidden weights	$\left[W_{H}^{T}\right]=\frac{1}{2}\times \left[W_{H}^{L}\right]$

Output bias	$\left[B_{O}^{T}\right]=\left[B_{O}^{L}\right]+\frac{1}{2}\left[W_{O}^{L}\right]\left[1\right]$

Output weights	$\left[W_{O}^{T}\right]=\frac{1}{2}\times \left[W_{O}^{L}\right]$

**Table 4:** Summary of analytic AF.

Ref.	Method	Convergence	Precision	Computational costs	Notes
[26]	Hyperbolic tangent	N/A	MSE = 0.0165	0.3435 us (Pentium II Machine)	

[16]	Arctangent	24 Epochs	MSE = 1*e* − 3	0.3435 us (Pentium II Machine)	Backpropagation with *η* = 0.5 (learning rate) and *α* = 0.8 (Momentum)

[18]	Quadratic sigmoid	4000 Epochs	MSE = 0.1–0.5 (2x more accurate than sigmoid on the same problem)	N/A	Backpropagation with *η* = 0.1 (learning rate) and *α* = 0.1 (Momentum), reduced during training

[19, 22]	Logarithmic-Exponential	250 Epochs	MSE = 0.048 (fitting problem) 97.2% accuracy (classification problem)	6.1090 us (Pentium II Machine)	

[20]	Spline-interpolant	2000 Epochs	MSE 〈dB〉 = −17.94 dB (3.26 dB less than sigmoid on the same problem)	N/A	Backpropagation with *μ* = 0.8 (learning rate)

[21]	Hermite polyn.	N/A	98.5% accuracy (classification problem)	N/A	

[22, 23]	Neural	50 Epochs	97.6% accuracy (classification problem) MSE = 0.082 (prediction problem)	N/A	

[24]	Composite AF	900 Epochs	92.8% accuracy (classification problem)	N/A	Even distribution of Gaussian, sinusoidal, and sigmoid

[24, 26]	Wave	250 Epochs	MSE = 0.2465 (15x less accurate than sigmoid on the same problem)	0.3830 us (Pentium II Machine)	

[25]	CosGauss	20 Epochs	MSE = 1.0 (10x more accurate than sigmoid on the same problem)	N/A	Implemented on a cascade correlation network

[26]	Sinc	250 Epochs	MSE = 0.0132 (0.25x more accurate than tanh on the same problem)	104.3360 us (Pentium II Machine)	

[26]	PolyExp	250 Epochs	MSE = 0.1007 (6x less accurate than tanh on the same problem)	0.3840 us (Pentium II Machine)	

[26]	SinCos	250 Epochs	MSE = 0.0114 (0.7x more accurate than tanh on the same problem)	1.1020 us (Pentium II Machine)	

**Table 5:** Summary of fuzzy logic AF.

Ref.	Method	Convergence	Precision	Computational costs	Notes
[27, 31]	Fuzzy-tanh	20 Epochs (up to 4x faster than tanh on the same problem)	MAE = 0.039 (2.5x more accurate than tanh) 93% accuracy (classification problem, 1% more than a classic MLP)	N/A	

[28]	Type 2 Fuzzy	41 Epochs (5x faster than tanh on the same problem)	MAE = 0.35	N/A	Backpropagation with learning rate *α* = 0.25

[32]	Fuzzy-tanh 2	N/A	RMSE = 0.0116 (comparable to tanhon the same problem) 95–98% accuracy (classification problem, comparable to tanh on the same problem)	N/A	Trained with extreme machine learning algorithm

**Table 6:** Summary of adaptive strategies.

Ref.	Method	Convergence	Precision	Computational costs	Notes
[34]	Scalable sigmoid (NNAAF-1)	6960 Epochs	% error = 0.033	Training time: 2739 s	Learning rate 0.9

[34]	Sin-sigmoid (NNAAF-2)	8232 Epochs	% error = 0.045	Training time: 2080 s	Learning rate 0.9

[34]	Morlet wavelet (NNAAF-3)	10000 Epochs	% error = 0.097	Training time: 3046 s	Learning rate 0.2

[35]	Sigmoid-radial-sin	5250 Epochs	N-RMSE = 0.09301	N/A	Trained with Levenberg Marquardt Algorithm

[36]	Sin-sigmoid	5000–9000 Epochs	89.60–94.3% accuracy (classification problem)	N/A	Implemented on higher order NN (HONN)

[37]	Trainable AF	20000 Epochs	RMSE 〈dB〉 = −35 dB	N/A	

**Table 7:** Summary of Lookup-Table approximations.

Ref.	Method	Convergence	Precision	Computational costs	Notes
[39]	RA-LUT (tanh)	N/A	MSE = 0.0053	17.5 us on 50 MHz FPGA	Resources used: 1815 LC comb. 4 LC reg.

[39]	RA-LUT (Logsig)	N/A	MSE = 0.1598	17.5 us on 50 MHz FPGA	Resources used: 1617 LC comb. 4 LC reg.

[40]	RA-LUT (Tansig) + FPU	N/A	MSE = 0.0150	47 us on 50 MHz FPGA	Resources used: 6538 LE

[41]	Error-optimized LUT	N/A	Max. error = 0.0378	Propagation delay: 0.95 ns (2x faster than classic LUT approach)	Gate Count: 70 Area (*μ*m^2^): 695.22 (10x smaller than classic LUT approach)

[42]	Compact RA-LUT	N/A	Max. error = 0.0182	Propagation delay: 2.46 ns	Gate Count: 181 Area (*μ*m^2^): 780 (4.5x smaller than classic LUT approach)

[43]	Hybrid	11 Epochs	% error = 1.88 (normalized to Full-Precision Floating Point)	Propagation delay: 0.8 ns	Trained on-chip with Levenberg Marquardt Algorithm Area (*μ*m^2^): 309

[43]	LUT	16 Epochs	% error = 1.34 (normalized to Full-Precision Floating Point)	Propagation delay: 2.2 ns	Trained on-chip with Levenberg Marquardt Algorithm Area (*μ*m^2^): 19592

[43]	RA-LUT	12 Epochs	% error = 0.89 (normalized to Full-Precision Floating Point)	Propagation delay: 1.0 ns	Trained on-chip with Levenberg Marquardt Algorithm Area (*μ*m^2^): 901

**Table 8:** Summary of Piecewise Linear Approximations.

Ref.	Method	Convergence	Precision	Computational costs	Notes
[44]	Piecewise linear “VHDL-C”	N/A	MSE = 0.00049	213 clock cycles	Resources used: Flip Flop Slices: 1277 4 input LUTs: 3767 BRAMS: 4

[45]	“Bajger-Omondi” method	N/A	Absolute error: up to 10^−6^ for 128 pieces with 18-bit precision	N/A	

[46]	PWL approximation	N/A	N/A	Propagation delay: 1.834 ns (100 ns more than LUT approach)	Resources used: 4 input LUTs: 108 (79 less than LUT approach) Slices: 58 (44 less than LUT approach) Total gates: 1029 (329 less than LUT)

[47, 48]	A-Law	N/A	% error = 0.63 84% accuracy (classification problem)	Propagation delay: 3.729 ns	Resources used: Slices: 185 LUTs: 101 Total gates: 1653

[47, 49]	Alippi	N/A	% error = 1.11	Propagation delay: 3.441 ns	Resources used: Slices: 127 LUTs: 218 Total gates: 1812

[47, 50, 51]	PLAN	N/A	% error = 0.63 85% accuracy (classification problem)	Propagation delay: 4.265 ns	Resources used: Slices: 127 LUTs: 218 Total gates: 1812

**Table 9:** Summary of hybrid and higher order techniques.

Ref.	Method	Convergence	Precision	Computational costs	Notes
[52]	4th-order Taylor	N/A	From 99.68% to 45% accuracy (classification problem)	Full NN computation time: 1.7 ms	Resources used: Slices: 4438 Flip Flops: 2054 LUTs: 8225

[53]	5th-order Taylor	N/A	% error = 0.51	N/A	Resources used: Slices: 4895 Flip Flops: 4777 LUTs: 8820

[54, 55]	Hybrid with PWL and RA-LUT	N/A	Up to 6.80*e* − 4 for 404 elements	Elaboration time: 40 *μ*s on 50 MHz FPGA	Resources used: Slices: 12 4 Inputs LUT: 17 BRAM: 1

[54, 55]	Hybrid with PWL and combinatorial	N/A	Up to 2.28*e* − 3 for 404 elements	Elaboration time: 40 *μ*s on 50 MHz FPGA	Resources used: Slices: 12 4 inputs LUT: 17 BRAM: 0

[56]	High precision sigmoid/exponential	N/A	RMSE = 8.362*e* − 6 (sigmoid) RMSE = 6.493*e* − 6 (exponential)	Maximum operative frequency: 868.056 MHz	Resources used: (as low as) 43 LUTs 26 registers

[57]	PWL and optimized LUT	N/A	N/A	Propagation delay: 0.06 ns	Resources used: Number of gates: 35 Area (*μ*m^2^): 148

[39, 58]	Four-polynomial tanh “4PY-T”	N/A	MSE = 0.0039	Full NN computation (50 MHz FPGA) 142 *μ*s	

[39, 58]	Five-polynomial tanh “5PY-T”	N/A	MSE = 0.0018	Full NN computation (50 MHz FPGA) 174 *μ*s	

[39, 58]	Five-polynomial Logsig “5PY-L”	N/A	MSE = 0.0075	Full NN computation (50 MHz FPGA) 185 *μ*s	

[61]	Piecewise Quadratic Tanh “scheme 2”	N/A	MEA = 4.1*e* − 3	Throughput rate: 0.773 MHz	Resources used: Area (*μ*m^2^): 83559.17

[60]	Piecewise Quadratic Tanh “Gs”	33 Epochs	SE = 0.1 99.6 generalization capability	N/A	

[47, 61]	Zhang quadratic approximation	N/A	MEA = 7.7*e* − 3 % error = 1.10	Propagation delay: 3.9 ns	Resources used: Slices: 93 LUTs: 86 Total gates: 1169

[62]	Adjusted LUT (*0.02 max. errors*)	N/A	MEA = 0.0121	Propagation delay: 2.80 ns	Area (*μ*m^2^): 5130.78

[62]	Adjusted LUT (*0.04 max. errors*)	N/A	MEA = 0.0246	Propagation delay: 2.31 ns	Area (*μ*m^2^): 3646.83

## Appendix

This appendix contains a summarized description, in form of tables (see Tables 4–9), of the results in terms of convergence error and performance reported by the authors in their articles. Some terms and acronyms, used in Tables 4, 5, 6, 7, 8, and 9, are explained below.

- Ref.: Reference in bibliography
- Method: The name of the activation function, as given by the authors in their work
- Precision: Performance of the proposed method in terms of ability to yield a correct output
- Computational costs: Estimate of the heaviness of the proposed method
- Epoch: Definition changes from one training algorithm to another. It generally corresponds to a full dataset “sweep” of the training algorithm
- Learning rate: Damping factor applied to weights correction during training (mainly used for backpropagation algorithms)
- Momentum: Inertial factor applied to maintain the direction of weight changing during training (mainly used for backpropagation algorithms)
- MSE/RMSE/N-RMSE: Mean Squared Error, Root Mean Squared Error, and Normalized Root Mean Squared Error
- % error: Percent of error (relative to signal, if not indicated otherwise)
- MAE: Mean Absolute Error
- Classification problem: Problem where the ANN must assign a belonging class to a set of inputs. Error relative to these problems is usually given in terms of % of correctly classified inputs
- Fitting problem: Problem where the ANN must replicate a nonlinear relationship. Error is usually given in terms of MSE between desired and actual output
- Prediction problem: Similar to the fitting problem, but involving state-space models
- Computation delay: Time required for digital commutation of the implemented hardware (upper limit to the maximum operative frequency)
- Elaboration time: Time required to compute the operations for a single neuron implementing the AF with the relative method
- Full NN computation: Time required to compute a single sample by NN featuring neurons implementing the AF with the relative method.

## References

[1] Cybenko G. (1989). Approximation by superpositions of a sigmoidal function. *Mathematics of Control, Signals, and Systems*.

[2] Leshno M., Lin V. Y., Pinkus A., Schocken S. (1993). Multilayer feedforward networks with a nonpolynomial activation function can approximate any function. *Neural Networks*.

[3] Hornik K., Stinchcombe M., White H. (1989). Multilayer feedforward networks are universal approximators. *Neural Networks*.

[4] Hornik K. (1991). Approximation capabilities of multilayer feedforward networks. *Neural Networks*.

[5] Funahashi K.-I. (1989). On the approximate realization of continuous mappings by neural networks. *Neural Networks*.

[6] Castro J. L., Mantas C. J., Benítez J. M. (2000). Neural networks with a continuous squashing function in the output are universal approximators. *Neural Networks*.

[7] Jaeger H. (2002). *Tutorial on Training Recurrent Neural Networks, Covering BPPT, RTRL, EKF and the ‘Echo State Network’ Approach*.

[8] Jaeger H. (2007). Echo state network. *Scholarpedia*.

[9] Lin T., Horne B. G., Tiňo P., Giles C. L. (1996). Learning long-term dependencies in NARX recurrent neural networks. *IEEE Transactions on Neural Networks*.

[10] Rodan A., Tiňo P. (2011). Minimum complexity echo state network. *IEEE Transactions on Neural Networks*.

[11] Li D., Han M., Wang J. (2012). Chaotic time series prediction based on a novel robust echo state network. *IEEE Transactions on Neural Networks and Learning Systems*.

[12] Wilamowski B. M. (2009). Neural network architectures and learning algorithms. *IEEE Industrial Electronics Magazine*.

[13] Hornik K., Stinchcombe M., White H. (1990). Universal approximation of an unknown mapping and its derivatives using multilayer feedforward networks. *Neural Networks*.

[14] Haykin S. (2004). *Neural Networks: A Comprehensive Foundation*.

[15] Chen T., Chen H. (1995). Universal approximation to nonlinear operators by neural networks with arbitrary activation functions and its application to dynamical systems. *IEEE Transactions on Neural Networks*.

[16] Kamruzzaman J., Aziz S. M. A note on activation function in multilayer feedforward learning.

[17] Bilski J. The backpropagation learning with logarithmic transfer function.

[18] Chiang C.-C., Fu H.-C. A variant of second-order multilayer perceptron and its application to function approximations.

[19] Piȩkniewski F., Rybicki L. Visual comparison of performance for different activation functions in MLP networks.

[20] Campolucci P., Capparelli F., Guarnieri S., Piazza F., Uncini A. Neural networks with adaptive spline activation function.

[21] Ma L., Khorasani K. (2005). Constructive feedforward neural networks using Hermite polynomial activation functions. *IEEE Transactions on Neural Networks*.

[22] Isa I. S., Saad Z., Omar S., Osman M. K., Ahmad K. A., Sakim H. A. M. Suitable MLP network activation functions for breast cancer and thyroid disease detection.

[23] Wong K. W., Leung C. S., Chang S.-J. (2002). Use of periodic and monotonic activation functions in multilayer feedforward neural networks trained by extended Kalman filter algorithm. *Vision, Image and Signal Processing, IEE Proceedings*.

[24] Hara K., Nakayamma K. Comparison of activation functions in multilayer neural network for pattern classification.

[25] Lee S.-W., Moraga C. Cosine-modulated Gaussian activation function for hyper-hill neural networks.

[26] Efe M. Ö. (2008). Novel neuronal activation functions for feedforward neural networks. *Neural Processing Letters*.

[27] Soria-Olivas E., Martín-Guerrero J. D., Camps-Valls G., Serrano-López A. J., Calpe-Maravilla J., Gómez-Chova L. (2003). A low-complexity fuzzy activation function for artificial neural networks. *IEEE Transactions on Neural Networks*.

[28] Karaköse M., Akin E. Type-2 fuzzy activation function for multilayer feedforward neural networks.

[29] Jang J.-S. R., Sun C.-T., Mizutani E. (1997). *Neuro-Fuzzy and Soft Computing: A Computational Approach to Learning and Machine Intelligence*.

[30] Zadeh L. A. (1975). The concept of a linguistic variable and its application to approximate reasoning—I. *Information sciences*.

[31] Subasi A. (2006). Automatic detection of epileptic seizure using dynamic fuzzy neural networks. *Expert Systems with Applications*.

[32] Huynh H. T., Won Y. Extreme learning machine with fuzzy activation function.

[33] Segee B. E. Using spectral techniques for improved performance in artificial neural networks.

[34] Özbay Y., Tezel G. (2010). A new method for classification of ECG arrhythmias using neural network with adaptive activation function. *Digital Signal Processing*.

[35] Ismail A., Jeng D.-S., Zhang L. L., Zhang J.-S. (2013). Predictions of bridge scour: application of a feed-forward neural network with an adaptive activation function. *Engineering Applications of Artificial Intelligence*.

[36] Xu S. (2010). Data mining using higher order neural network models with adaptive neuron activation functions. *International Journal of Advancements in Computing Technology*.

[37] Wu Y., Zhao M., Ding X. Beyond weights adaptation: a new neuron model with trainable activation function and its supervised learning.

[38] Laudani A., Lozito G. M., Riganti Fulginei F., Salvini A. An efficient architecture for floating point based MISO neural neworks on FPGA.

[39] Lozito G.-M., Laudani A., Riganti-Fulginei F., Salvini A. (2014). FPGA implementations of feed forward neural network by using floating point hardware accelerators. *Advances in Electrical and Electronic Engineering*.

[40] Santos P., Ouellet-Poulin D., Shapiro D., Bolic M. Artificial neural network acceleration on FPGA using custom instruction.

[41] Zamanlooy B., Mirhassani M. (2014). Efficient VLSI implementation of neural networks with hyperbolic tangent activation function. *IEEE Transactions on Very Large Scale Integration (VLSI) Systems*.

[42] Meher P. K. An optimized lookup-table for the evaluation of sigmoid function for artificial neural networks.

[43] Leboeuf K., Muscedere R., Ahmadi M. Performance analysis of table-based approximations of the hyperbolic tangent activation function.

[44] Braga A. L., Llanos C. H., Göhringer D., Obie J., Becker J., Hübner M. Performance, accuracy, power consumption and resource utilization analysis for hardware/software realized artificial neural networks.

[45] Bajger M., Omondi A. (2008). Low-error, high-speed approximation of the sigmoid function for large FPGA implementations. *Journal of Signal Processing Systems*.

[46] Saichand V., Nirmala D. M., Arumugam S., Mohankumar N. FPGA realization of activation function for artificial neural networks.

[47] Tisan A., Oniga S., Mic D., Buchman A. (2009). Digital implementation of the sigmoid function for FPGA circuits. *Acta Technica Napocensis—Electronics and Telecommunications*.

[48] Myers D. J., Hutchinson R. A. (1989). Efficient implementation of piecewise linear activation function for digital VLSI neural networks. *Electronics Letters*.

[49] Alippi C., Storti-Gajani G. Simple approximation of sigmoidal functions: realistic design of digital neural networks capable of learning.

[50] Amin H., Curtis K. M., Hayes-Gill B. R. (1997). Piecewise linear approximation applied to nonlinear function of a neural network. *IEE Proceedings—Circuits, Devices and Systems*.

[51] Tisan A., Oniga S., Gavrincea C. Hardware implementation of a MLP network with on-chip learning.

[52] Lee Y., Ko S.-B. FPGA implementation of a face detector using neural networks.

[53] Khodja D. E., Kheldoun A., Refoufi L. Sigmoid function approximation for ANN implementation in FPGA devices.

[54] Sartin M. A., Da Silva A. C. R. Approximation of hyperbolic tangent activation function using hybrid methods.

[55] Sartin M. A., da Silva A. C. (2014). ANN in hardware with floating point and activation function using hybrid methods. *Journal of Computers*.

[56] Ortega-Zamorano F., Jerez J. M., Juarez G., Perez J. O., Franco L. High precision FPGA implementation of neural network activation functions.

[57] Saranya S., Elango B. Implementation of PWL and LUT based approximation for hyperbolic tangent activation function in VLSI.

[58] Lozito G. M., Bozzoli L., Salvini A. Microcontroller based maximum power point tracking through FCC and MLP neural networks.

[59] Lin C.-W., Wang J.-S. A digital circuit design of hyperbolic tangent sigmoid function for neural networks.

[60] Kwan H. K. (1992). Simple sigmoid-like activation function suitable for digital hardware implementation. *Electronics Letters*.

[61] Zhang M., Vassiliadis S., Delgado-Frias J. G. (1996). Sigmoid generators for neural computing using piecewise approximations. *IEEE Transactions on Computers*.

[62] Namin A. H., Leboeuf K., Muscedere R., Wu H., Ahmadi M. Efficient hardware implementation of the hyperbolic tangent sigmoid function.

[63] Carrasco M., Mancilla-David F., Fulginei F. R., Laudani A., Salvini A. (2013). A neural networks-based maximum power point tracker with improved dynamics for variable dc-link grid-connected photovoltaic power plants. *International Journal of Applied Electromagnetics and Mechanics*.

[64] Mancilla-David F., Riganti-Fulginei F., Laudani A., Salvini A. (2014). A neural network-based low-cost solar irradiance sensor. *IEEE Transactions on Instrumentation and Measurement*.

[65] Riganti-Fulginei F., Laudani A., Salvini A., Parodi M. (2013). Automatic and parallel optimized learning for neural networks performing MIMO applications. *Advances in Electrical and Computer Engineering*.

[66] Fulginei F. R., Salvini A., Parodi M. (2012). Learning optimization of neural networks used for MIMO applications based on multivariate functions decomposition. *Inverse Problems in Science and Engineering*.

[67] LeCun Y., Denker J. S., Solla S. A. (1989). Optimal brain damage. *Advances in Neural Information Processing Systems (NIPs)*.

[68] Hassibi B., Stork D. G. (1993). *Second Order Derivatives for Network Pruning: Optimal Brain Surgeon*.

[69] Prechelt L., Fakultat Fur Informatik (1994). Proben1: a set of neural network benchmark problems and benchmarking rules.

